# Aspergoterpenins A–D: Four New Antimicrobial Bisabolane Sesquiterpenoid Derivatives from an Endophytic Fungus *Aspergillus versicolor*

**DOI:** 10.3390/molecules23061291

**Published:** 2018-05-28

**Authors:** Zhi-Yong Guo, Ming-Hui Tan, Cheng-Xiong Liu, Meng-Meng Lv, Zhang-Shuang Deng, Fei Cao, Kun Zou, Peter Proksch

**Affiliations:** 1 Hubei Key Laboratory of Natural Product Research and Development, College of Biological and Pharmaceutical Sciences, China Three Gorges University, Yichang 443002, China; zhyguoctgu@foxmail.com (Z.-Y.G.); moonshinetan@sina.com (M.-H.T.); liuchengxiong666@126.com (C.-X.L.); mengmenglv828@yeah.net (M.-M.L.); kzou@ctgu.edu.cn (K.Z.); 2 Hubei Engineering Research Center for Three Gorges Regional Plant Breeding, China Three Gorges University, Yichang 443002, China; 3 College of Pharmaceutical Sciences, Key Laboratory of Pharmaceutical Quality Control of Hebei Province, Key Laboratory of Medicinal Chemistry and Molecular Diagnostics of Education Ministry of China, Hebei University, Baoding 071002, China; 4 Institute of Pharmaceutical Biology and Biotechnology, Heinrich-Heine-Universität Düsseldorf, Universitätsstrasse 1, 40225 Düsseldorf, Germany; Peter.Proksch@uni-duesseldorf.de

**Keywords:** bisabolanese squiterpenoid, endophytic fungus, *Aspergillusversicolor*, structural elucidation, antimicrobial activities

## Abstract

Aspergoterpenins A–D (**1**–**4**), four new bisabolane sesquiterpenoid derivatives, were obtained from the endophytic fungus, *Aspergillus versicolor*, together with eight known compounds (**5**–**12**), and their structures were elucidated by a comprehensive analysis of their NMR (Nuclear Magnetic Resonance), MS (Mass Spectrum) and CD (Circular Dichroism) spectra. Aspergoterpenin A (**1**) was the first example with a characteristic ketal bridged-ring part in the degraded natural bisabolane-type sesquiterpene structures. The compounds **1**–**12** displayed no significant activities against four cancer cell lines (A549, Caski, HepG2 and MCF-7). Further, the antimicrobial activities to *Erwinia carotovora* sub sp. Carotovora were evaluated, and the results showed that compounds **1**–**12** displayed antimicrobial activities with MIC values ranging from 15.2 to 85.2 μg/mL.

## 1. Introduction

Soft rot is the main disease threatening the economical crop, *Amorphophallus rivieri* Durieu [1], which is called the “cancer” of *Amorphophallus rivieri* Durieu. The soft rot pathogen was identified as *Erwinia carotovora* sub sp. Carotovora [2].After the soft rot pathogens penetrated the leaves of *Amorphophallusrivieri*, and the leaves and tubers began to rot and stink, which led the tubers yield to reduce the production from 30% to 80% and sometimes even to total crop failture [3]. Another feature of the soft rot pathogen is strong infectivity. Thus, the soft rot pathogen is an aporia and a bottle-neck for developing the industrial chain of *Amorphophallus rivier* in China [4]. The fungal genus of Aspergillus was widely distributed in soil, plant and ocean environments, and attracted the attention of chemists and pharmacologists because of their enormous metabolites production potential and multifariously biological activities [5,6,7,8,9,10,11,12,13,14]. The investigation into the chemical structure and bioassay diversities of the endophytic fungi around the Three Gorges area were performed, we found that the crude extract of the endophytic fungus, *Aspergillus versicolor* (No. 65), displayed moderate inhibition activity against *Erwinia carotovora* sub sp. Carotovora. Therefore, thiscontinuous chemical investigation resulted in the acquisition of twelve bisabolane sesquiterpenoid derivatives, and their structural elucidations and antimicrobial activities were reported in this paper.

## 2. Results and Discussion

Aspergoterpenin A (**1**) was obtained as colorless oil, $[\alpha ]{}_{D}^{25}$ = −40.0 (c = 0.08, MeOH). UV/Vis (MeOH): *λ*_max_ (log *ε*_max_) = 242 (3.60) nm, 301 (2.40) nm, IR (film) *υ_max_*: 3225, 2996, 1708, 1600, 1498, 1450, 1375 cm^−1^. Its molecular formula was established as C_14_H_16_O_4_ by HR-ESI-MS by the pseudomolecular ion peak at *m/z* 249.1122 [M + H]^+^ (cald. 249.1127 for C_14_H_17_O_4_). According to the formula of compound **1**, there were seven degrees of unsaturation in **1**. In the ^1^H-NMR spectrum, three groups of olefinic proton signals lied at *δ* 7.16 (d, 8.0, 1H), 7.50 (dd, 1.7, 8.0, 1H) and 7.34 (d, 1.7, 1H) (see Table 2). In consideration of the chemical shifts and coupling constants of the above protons signals, there was a 1,2,4-trisubstituented phenyl ring in compound **1**, which was confirmed by the correlations from H-3 (*δ* 7.16) to H-4 (*δ* 7.50) and from H-4 (*δ* 7.50) to H-6 (*δ* 7.34) in the ^1^H-^1^H COSY spectrum. Furthermore, there were two singlet methyl groups at *δ* 1.50 (s, 3H) and 1.60 (s, 3H), and the remaining proton signals were evenly distributed between *δ* 1.45 (m, 1H) and *δ* 1.97 (br d, 13.3, 1H). In the ^13^C and DEPT (Distortionless Enhancement by Polarization Transfer) spectra, fourteen carbon resonance signals were observed, including a carbonyl group at *δ* 169.7 (C-7), six olefinic carbon resonance peaks at *δ* 154.8 (C-1), 132.8 (C-2), 131.9 (C-5), 125.2 (C-3), 121.9 (C-4), 117.0(C-6), one ketal resonance signal at *δ*100.9 (C-12), one oxygenated carbon signal at *δ* 74.9 (C-8), three methylene peaks at *δ* 38.1 (C-9), 36.5 (C-11) and 18.3 (C-10), two methyl groups peaks at *δ* 28.6 (C-13) and 26.9 (C-14) (see Table 1). These observations were consistent with the results of the proton NMR spectrum of **1**.

**Figure 1:**
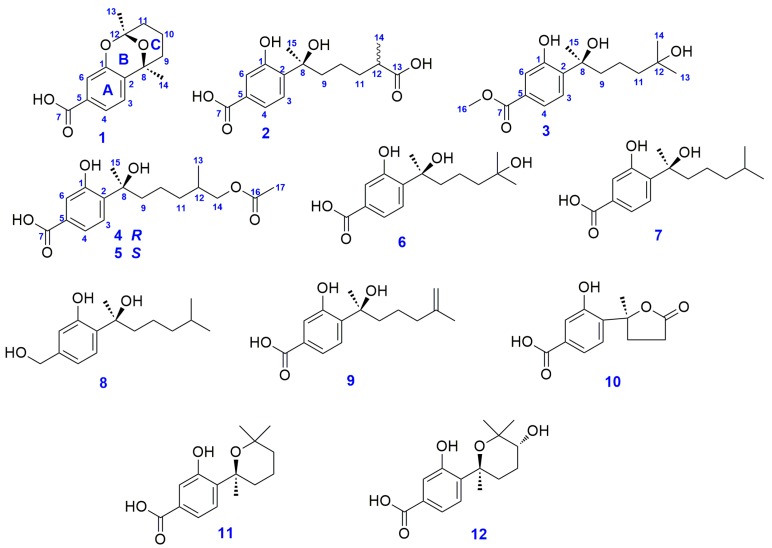
Thestructures of compounds **1**–**12**.

**Figure 2:**
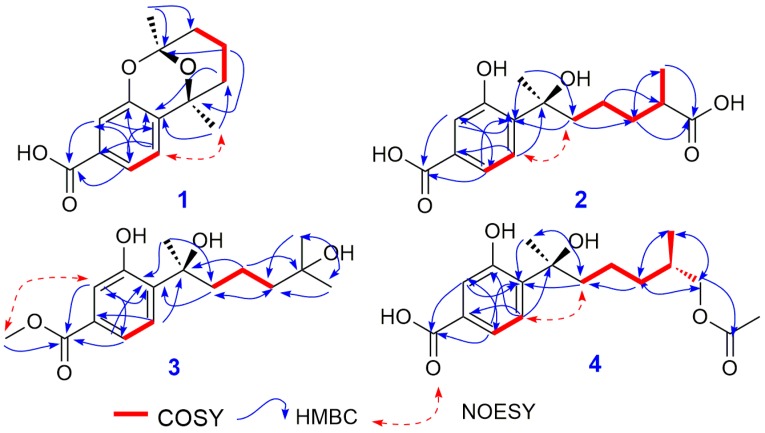
The COSY and key HMBC NMR correlations of compounds **1**–**4**.

The comprehensive analysis of the ^1^H-^1^H COSY, HSQC and HMBC NMR spectra resulted in the establishment ofthe complete structure of compound **1**. In the ^1^H-^1^H COSY spectrum of compound **1**, the correlations from the proton at *δ* 1.81 (H-9a) to *δ* 1.62 (H-10a), and from the hydrogen signal at *δ* 1.45 (H-10b) to *δ* 1.97 (H-11a), suggested that there was a -CH_2_CH_2_CH_2_- partial structure in compound **1**, which was proved by the HMBC correlations of H-10, H-11a/C-9, H-9,and H-11a/C-10. In the HMBC spectrum of **1**, the correlations of H-3/C-1, C-6, H-4/C-2, C-3, C-6, H-6/C-1 and C-4 demonstrated the existence of the benzene ring in **1**, which was consistent with the results of ^1^H-^1^H COSY spectrum. The cross peaks from H-4 and H-6 to C-7 proved that the carbonyl group at *δ* 169.7was connected to the benzene ring by C-5. The correlations of H-3/C-8, H-9, H-14/C-2, H-9, H-10, H-13, H-14/C-7, H-10, H-11 and H-13/C-12 revealed the presence of C ring in **1** (see Figure 1 and Figure 2). Finally, the B ring was authenticated by the molecular weight of 248, signifying a ketal function group, rather thana semi-ketal group in compound **1**. Therefore, the planar structure of **1** was constructed on the basis of an extensive analysis of the NMR spectra of **1** (see Figure 1).

**Figure 3:**
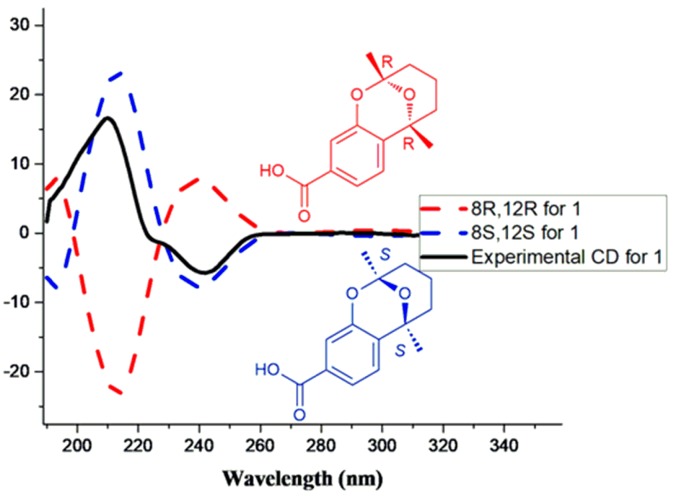
The calculated ECD and experimental CD spectra of compound **1**.

In the NOESY spectrum, there was no key correlation between H-13 (*δ* 1.50) and H-14 (*δ* 1.60), and the CD spectrum showed a positive Cotton effect at 209 nm with Δε +18 (mdeg) and a negative Cotton effect at 240 nm with Δ*ε*−6 (mdeg). Then, the ECD of compound **1** was calculated using the TDDFT (Time-Dependent Density Functional Theory) method on the computer. Comparing to the theoretical calculation of theECD spectrum andthe experimental CD spectrum (see Figure 3), we found that the experimental CD spectrum agreed with that of 8*S* and 12*S* enantiomer. Thus, the absolute configurations of C-8 and C-12 were determined as 8*S* and 12*S*. The structure of compound **1** was retrieved in the references data, and compound **1** was found to be an unreported compound, named aspergoterpeninA (**1**).

Structurally, aspergoterpeninA (**1**) belonged to a degraded bisabolane-type sesquiterpene. Furthermore, aspergoterpeninA (**1**) bore a characteristic bridged-ring structure, which was formed by a carbonyl group reacting toone phenolic hydroxyl group and an alcohol hydroxyl group. Aspergoterpenin A (**1**) was the first example with a ketal bridged-ring characteristic structure in the degraded bisabolane-type sesquiterpenes.

Aspergoterpenin B (**2**) was obtained as the colorless oil, $[\alpha ]{}_{D}^{25}$ = −35.0 (c = 0.03, MeOH). UV/Vis (MeOH): *λ*_max_ (log *ε*_max_) = 249 (3.60) nm, 303 (2.40) nm, IR (film) *υ*_max_: 3215, 2990, 1689, 1590, 1500, 1456, 1375 cm^−1^. Its molecular formula of C_15_H_20_O_6_ was confirmed with HR-ESI-MS by the pseudomolecular ion peak at *m/z* 319.1151 [M + Na]^+^ (cald. 319.1152 for C_15_H_20_O_6_Na), and six degrees of unsaturation appeared in compound **2**. The ^1^H-NMR spectrum of compound **2** was similar to that of the known compound, (−)-hydroxsydonic acid (**6**) [15], a bisabolane-type sesquiterpene metabolite from Aspergillus genus. The differences between compound **2** and **6** in the proton nuclear magnetic resonance spectra were that two additional proton signals appeared at *δ* 2.34 (m, 1H) and 1.07 (d, 7.0, 3H) (see Table 2), and the doublet methyl group (*δ* 1.07) in compound **2** displaced the two singlet methyl groups in compound **6**. The remaining proton signals were exactly the same. Comparing the carbon NMR data of compound **2** and **6**, we found that there was an additional carboxyl group signal appearing at *δ* 181.6 and one methyl group at *δ* 17.7 in compound **2**, displacing the corresponding two methyl groups at *δ* 28.8 and 29.1 in compound **6** (see Table 1).

Further analysis of the ^1^H-^1^H COSY, HSQC and HMBC spectra of **2**, in the ^1^H-^1^H COSY spectrum, the cross peaks of H-14 [1.07 (d, 7.0, 3H)]/H-12 [2.34 (m, 1H)]/H-11 [1.58 (m, 1H), 1.34 (m, 1H)] implied that there was a subunit structure of -CH_2_-CH(R)-CH_3_in compound **2**, and the above COSY deductions were verified by the correlations of H-11/C-12, H-12/C-14, H-14/C-11 and C-12 in the HMBC spectrum of **2**. Furthermore, the proton signals of H-11, H-12 and H-14 correlated to the C-13 at *δ* 181.6, which indicated that the R group in the subunit structure of -CH_2_-CH(R)-CH_3_ was a carboxyl group in **2**. The remaining structure of **2** was the same as that of compound **6**, according to the HMBC correlations in compound **2**.

**Figure 4:**
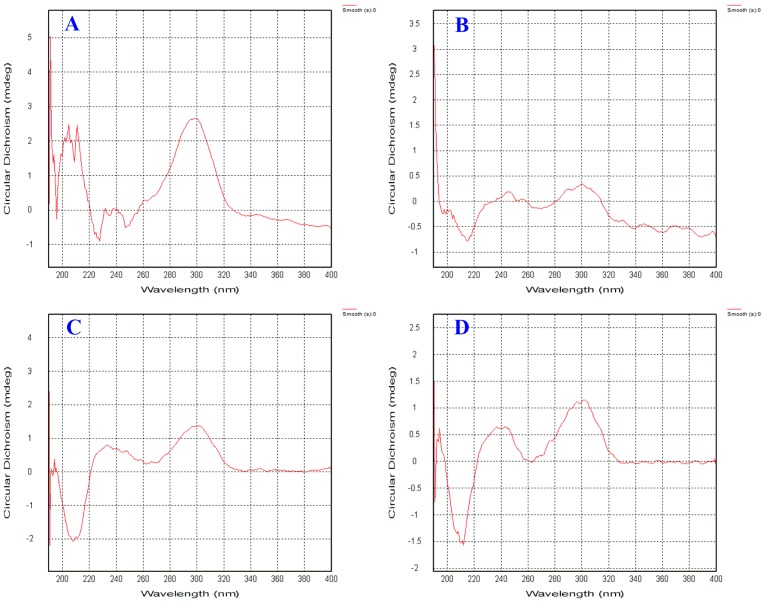
The CD spectra of compounds **2**–**5**. (**A** for compound **2**, **B** for compound **3**, **C** for compound **4** and **D** for compound **5**).

In compound **2**, there were two chiral centers at C-8 and C-12, and the absolute configuration of C-8 was inferred as the *S* configuration by comparing the CD spectra of compound **2** with that of compound **6** (see Figure 4). The configuration of C-12 was not determined by calculating the ECD spectrum usingthe TDDFT method. Finally, compound **2** was identified as a new compound with the name of aspergoterpenin B, as shown in Figure 1.

Aspergoterpenin C (**3**) was obtained as colorless oil, $[\alpha ]{}_{D}^{25}$ = −10.5 (c = 0.07, MeOH). UV/Vis (MeOH): *λ*_max_ (log *ε*_max_) = 250 (3.60), 305 (2.40) nm, IR (film) *υ*_max_: 3210, 2989, 1705, 1675, 1604, 1485, 1445, 1380, 1365 cm^−1^. Its molecular formula of C_16_H_24_O_5_ was determined by HR-ESI-MS with the pseudomolecular ion peak at *m/z* 319.1523 [M + Na]^+^ (cald. 319.1521 for C_16_H_24_O_5_Na). Comparing the ^1^H and ^13^C-NMR data of compound **3** with that of (−)-hydroxsydonic acid (**6**), except that an additional methoxyl group at *δ* 3.88 (s,3H) in ^1^H-NMR spectrum, and an additional carbon signal at *δ* 52.5 in ^13^C-NMR spectrum, the rest proton and carbon signals of compound **3** were exactly same as those of (−)-hydroxsydonic acid (**6**), The key correlation from *δ* 3.88 (s,3H) (H-16) to *δ* 168.5 (C-7) indicated that the methoxyl group was connected to the carboxyl group by an ester rather than an ether function group. By comparing the CD spectra of compound **3** with that of (−)-hydroxsydonic acid (**6**) (see Figure 4), the absolute configuration of the chiral carbon (C-8) was revealed as 8*S*. Compound **3** was the new natural product, named aspergoterpenin C.

Aspergoterpenin D (**4**) was obtained as colorless oil, $[\alpha ]{}_{D}^{25}$ = −15.0 (c = 0.05, MeOH). UV/Vis (MeOH): *λ*_max_ (log *ε*_max_) = 250 (3.80), 304 (2.10) nm, IR (film) *υ*_max_: 3220, 2990, 1689, 1589, 1500, 1490, 1375 cm^−1^.Its molecular formula was established as C_17_H_24_O_6_ by HR-ESI-MS with the pseudomolecular ion peak at *m/z* 325.1565 [M + H]^+^ (cald. 325.1561 for C_17_H_2__5_O_6_). During the purification and NMR measurement of **4**, we found that there were four lower strength carbon signals at *δ* 70.53 (high)/70.45 (lower), 36.66 (lower)/36.61 (high), 28.92 (high)/28.83 (lower) and 17.13 (lower)/17.06 (high) in the ^13^C-NMRspectrum, and we doubted that there were two epimers in the first purification process of **4**. Therefore, the chiral HPLC column was employed to purify the epimer mixtures, and compounds **4** and **5** were obtained. The NMR data of compounds **4** and **5** were almost the same. Comparing the NMR data of compound **5** withthe references’ NMR data, compound **5** shared the same NMR data as (7*S*,11*S*)-(+)-12-acetoxysydonic acid [16]. Thus, compound **5** was identified as (7*S*,11*S*)-(+)-12-acetoxysydonic acid. However, compound **4** showed little difference to compound **5** in carbon NMR data. Comparing the CD spectra of **4** and **5**, we found that they shared the same Cotton effect at the same wavelength in 200, 240 and 340 nm. From the structure of compound **4** (see Figure 1), the first chiral carbon of C-8 was adjacent to the benzenic chromophore, whereas, the chiral carbon of C-12 was separated by a three -CH_2_- chain. While, the chromophore of the acetyl group displayed poor chromaticity performance, which led to a weak Cotton effect at the shorter wavelength range, or overlapped with the Cotton effect induced by the C-8 in the CD spectrum of compound **4**. The CD spectrum of compound **4** shared the same variation tendency as (−)-hydroxsydonic acid (**6**), thus, the absolute configuration of C-8 was *S*, and the absolute configuration of C-12 was deduced as *R*, according to the chiral HPLC results and the reference data. Compound **4** was found to be a new natural product, named aspergoterpenin D.

The known compounds **6**–**12** were identified as 11-hydroxysydonic acid [15] (**6**), (*S*)-(+)-sydonic acid (**7**) [15,17], (−)-sydonol (**8**) [18], (*S*)-(+)-11-dehydrosydonic acid (**9**) [16], 1-hydroxyboivinianic acid (**10**) [19], sydowic acid (**11**) [15,20], and (7*R*,10*S*)-10-hydroxysydowic acid (**12**) [20] by comparing their NMR data and CD spectra with the reference data.

The isolated compounds **1**–**12** were evaluated to four cancer cell lines (A549, Caski, HepG 2 and MCF-7) with IC_50_ values > 50 μg/mL, and displayed no significant activities. Further, the antimicrobial activities against *Erwinia carotovora* sub sp. Carotovora were evaluated, and the results showed that compounds **1**–**12** displayed antimicrobial activity with MIC (Mininum Inhibitory Concentration) values ranging 15.2 to 85.2 μg/mL (see Table 3).

## 3. Materials and Methods

### 3.1. General Experimental Procedures

The UV spectrum was obtained on a SCINCO Spectrometer, and IR spectrums were recorded on a Nicoler Auatar Spectrometer series FT360 spectrophotometer. NMR spectra (1D and 2D) were recorded on a Bruker Ultrashield–400 MHz NMR spectrometer (Karlsruhe, Germany). Mass spectra were obtained on an Agilent 6210 ESI (Electrospray Ionization)/TOF (Time of Flight) MS spectrometer (Palo-Alto, CA, USA), to obtain HRMS spectra, and on an EIMS (Electron Ion Mass Spectrum) Thermofisher ISQ spectrometer. Semi-preparative HPLC was performed on a Dionex Ultra-3000 (Sunnyvale, CA, USA) and a Waters 1525 (Milford, MA, USA) using a Cosmosil C-18 column (10 μm × 20 mm × 250 mm and 5 μm × 4.6 mm × 250 mm). CD spectra were measured on a qCD spectrometer [APL (Applied Photophysics Limited Company, Leatherhead, UK)]. Silica gel GF_254_ (10–40 μm), prepared for TLC (Thin Layer Chromatography), and silica gel (200–300 mesh), for column chromatography (CC), were obtained from Qingdao Marine Chemical Factory (Qingdao, People’s Republic of China). Fractions were monitored by TLC and the spots were visualized under aultraviolet lamp at 254 nm and in an iodine cylinder.

### 3.2. Isolation and Identification of the Endophytic Fungus

The healthy leaves of *Elaeocarpus decipiens* Hemsl were collected at Cuiping Hill on the campus of China Three Gorges University. The leaves were washed underrunning water and immersed in 5–10% NaClO solution for 5 min, and thenin 75% ethanol/water solution for 3 min. Finally, they were washed withaseptic water three times and removed from the aseptic water usingstrile filter papers. The sterilized leaves were cut into small pieces with the size of 0.5 × 0.5 cm^2^, and those pieces were placed on the PDA (Potato Dextrose Agar) plates in the incubator at 28 °C. After the fungi on the PDA plates grew sufficiently for further purification, and repeatedly purified by the Streak method, until single colonies were obtained. The pure single colonies were deposited on the slants at 4 °C. In total, the five endophytic fungi were obtained from the leaves of *Elaeocarpus decipiens* Hemsl, and those fungi were fermented with 200 mL of PDB (Potato Dextrose Broth) liquid medium in 500 mL Erlenmeyer flasks. The fungus *Aspergillus versicolor* (No. 65) was selected for further chemical constituents investigation according to the results of antimicrobial biological activity assays and chemical structural diversity analysis by the HPLC (High Performance Liquid Chromatography)–DAD (Diode Array Detector) method. The *Aspergillus versicolor* (No. 65) was cultured on a solid rice medium (140 g rice + 70 g bran + 200 mL tap water) in ten 3000 mL Erlenmeyer flasks at room temperature for 40 days. The plant samples were identified as *Elaeocarpus decipiens* Hemsl by Prof. Yubing Wang, and the samples were disposed in Hubei Key Laboratory of Natural Product Research and Development (Yichang, China Three Gorges University) with the voucher specimen No. 20150828B. During the identification fungus No. 65, it was found that the 18*S*rDNA sequence results shared 99.8% similarity with the *Aspergillus versicolor*, thus, the fungus of No. 65 was identified as *Aspergillus versicolor*.

### 3.3. Extraction and Isolation of Compounds

The solid rice medium with the growing endophytes was crashedand extracted three times with 10 L ethyl acetate at room temperature for two weeks. Then, the extract was concentrated in avacuum to yield 8 g of crude extract. The 6 g of crude extract was subjected to silica gel column chromatography and eluted from light petroleum ether to acetyl ester, and then to methanol. The subfractions were merged into eight fractions, according to the TLC results. The fraction B (0.6 g) was further subjected to silica gel column chromatography and to C-18 reverse phase silica gel column chromatography, with a gradient elution from 25% to 90% CH_3_CN-H_2_O (containing 0.02% acetic acid), to obtain compound **1** (2 mg), compound **11** (4 mg) and compound **6** (500 mg). The subfraction C-3 (0.3 g) was further purified by semi-preparative reverse-phase HPLC with 45% CH_3_CN-H_2_O (containing 0.02% acetic acid) isocratic elution to obtain compound **7** (*t_R_* 26.8 min, 3.0 mg) and compound **8** (*t_R_* 46.5 min, 1.5 mg). The subfraction C-4 (0.8 g) was purified by silica gel column chromatography and semi-preparative HPLC, with gradient elution from 40% to 56% MeOH-H_2_O (containing 0.02% acetic acid), to obtain compound **9** (*t_R_* 32.5 min, 3.0 mg). The subfraction E (2 g) was subjected to SephadexLH-20 chromatography with MeOH, and silica gel column chromatography, and further purified with by HPLC with CH_3_CN-H_2_O elution system (containing 0.02% acetic acid) from 34% to 50%, to obtain compound **12** (*t_R_* 16.4 min, 3.0 mg), compound **3** (*t_R_* 25.9 min, 2.0 mg) and the mixture of **4** and **5**. Finally, the mixture was further purified with 15% alcohol-hexane (containing 0.02% acetic acid)on a chiral HPLC column, to obtain compound **4** (*t_R_* 25.8 min, 4.0 mg) and **5** (*t_R_* 38.8 min, 3.0 mg). The subfraction F (500mg) was further purified with semi-preparative HPLC and CH_3_CN-H_2_O elution system (containing 0.02% acetic acid) from 30% to 40% to obtain compound **10** (*t_R_* 16.4 min, 3.5 mg) and compound **2** (*t_R_* 27.2 min, 2.0 mg).

### 3.4. Computational Methods

A systematic conformational search of compound **1** was carried out by the conflex program with aMMFF94 (Merk Molecular Force Field) force field. All of the conformers were further optimized by the B3LYP/6-311G (d,p). The same level of harmonic vibrational frequencies was calculated to confirm their stability and to estimate their relative Gibbs free energies at 298.15 K. The ECD of compound **1** wascalculated using the TDDFT method at the PCM/PBE0/6-311 + +G (d,p) level. The number of singlet excited states per conformer was 40. The specific rotation of the hexaricins was calculated with the DFT (Density Funtional Theory) method at the PCM/B3LYP/6-311 + +G (d,p) level. Solvent effects were considered by the Integral Equation Formalism Polarizable Continuum Model (IEFPCM) in MeOH. Finally, the ECDs and the optical rotation of the conformers were Boltzmann-weighted according to the calculated Gibbs free energies. The calculated ECD curves were generated using SpecDis1.64, with σ = 0.26 ev and the UV shift at 10 nm. All DFT calculations were performed using the Gaussian09 program.

### 3.5. Biological Activity Assay

#### 3.5.1. Cytotoxic Activity to Four Cancer Cells In Vitro

A549, Caski, HepG2 and MCF-7 cells were cultured in a RPMI (Roswell Park Memorial Institute) 1640 medium (HyClone) supplemented with 10% FBS (fetal bovine serum) (HyClone). The cells were maintained in 5% CO_2_ at 37 °C. The 3-(4,5-Dimethyl thiazol-2-yl)-2,5-diphenyl-2*H*-tetrazolium bromide (MTT, Sigma, St. Louis, MO, USA) colorimetric assay was used to evaluate cell proliferation in the presence of different chemicals. The cells were seeded in 96-well culture plates and treated with vehicles or desired concentrations of chemicals for a further 24 h. After treatment, cells were incubated at 37 °C with MTT (10 μL/well, 5 mg/mL) for 4 h, and the cell growth response to the chemicals was determined by measuring the absorbance at 570 nm on a plate reader. Three replicates were used for each treatment. In the cytotoxic activity in vitro experiment, mitomycin was employed as a positive control for the cytotoxic activity assay.

#### 3.5.2. Antimicrobial Activity

Antimicrobial assay against the plant-pathogenic *Erwinia carotovora* sub sp. Carotovora was carried out using the well diffusion method. Amphotericin B wasused as a positive control for the antifungal assay [21].

### 3.6. Spectroscopic Data of the Compounds

Aspergoterpenin A (**1**), colorless oil; $[\alpha ]{}_{D}^{25}$ = −40.0 (c = 0.08, MeOH). UV/Vis (MeOH): *λ*_max_ (log *ε*_max_) = 242 (3.60) nm, 301 (2.40) nm, IR (film) *υ*_max_: 3225, 2996, 1708, 1600, 1498, 1450, 1375 cm^−1^.HRESIMS: *m/z* 249.1122 [M + H]^+^ (Cald. 249.1127 for C_14_H_16_O_4_), NMR data (see Table 1 and Table 2).

Aspergoterpenin B (**2**), colorless oil; $[\alpha ]{}_{D}^{25}$ = −35.0 (c = 0.03, MeOH). UV/Vis (MeOH): *λ*_max_ (log *ε*_max_) = 249 (3.60) nm, 303 (2.40) nm, IR (film) *υ_max_*: 3215, 2990, 1689, 1590, 1500, 1456, 1375 cm^−1^; HRESIMS *m/z* 319.1151 [M + Na]^+^ (cald. 319.1152 for C_15_H_20_O_6_Na), NMR data (see Table 1 and Table 2).

Aspergoterpenin C (**3**), colorless oil; $[\alpha ]{}_{D}^{25}$ = −10.5 (c = 0.07, MeOH). UV/Vis (MeOH): *λ*_max_ (log *ε*_max_) = 250 (3.60), 305 (2.40) nm, IR (film) *υ*_max_: 3210, 2989, 1705, 1675, 1604, 1485, 1445, 1380, 1365 cm^−1^; HRESIMS *m/z* 319.1151 [M + Na]^+^ (cald. 319.1152 for C_15_H_20_O_6_Na), NMR data (see Table 1 and Table 2).

Aspergoterpenin D (**4**), colorless oil; $[\alpha ]{}_{D}^{25}$ = −15.0 (c = 0.05, MeOH). UV/Vis (MeOH): *λ*_max_ (log *ε*_max_) = 250 (3.80), 304 (2.10) nm, IR (film) *υ*_max_: 3220, 2990, 1689, 1589, 1500, 1490, 1375 cm^−1^; HRESIMS *m/z* 325.1565 [M + H]^+^ (cald. 325.1561 for C_17_H_2__5_O_6_), NMR data (see Table 1 and Table 2).

## 4. Conclusions

Twelve bisabolane sesquiterpenoid derivatives **1**–**12**, including four new compounds, aspergoterpenins A–D (**1**–**4**), were obtained from the endophytic fungus, *Aspergillus versicolor* (No. 65), and their structures were elucidated by a comprehensive analysis of their NMR, MS and CD spectra.Aspergoterpenin A (**1**) was the first example with a ketal bridged-ring characteristic structure in the degraded bisabolane-type sesquiterpenes. The obtained compounds **1**–**12** displayed no significant activities against four cancer cell lines (A549, Caski, HepG2 and MCF-7) with IC_50_ values >50 μg/mg. The antimicrobial activities against *Erwinia carotovora* sub sp. Carotovora were evaluated and the results showed that compounds **1**–**12** displayed antimicrobial activities with MIC values ranging from 15.2 to 85.1 μg/mL.

## Figures and Tables

**Table 1:** The ^13^C-NMR data of compounds **1**–**4** (100 MHz, CD_3_OD-*d_4_*).

No.	1	2	3	4
1	154.8 (C)	156.8 (C)	157.0 (C)	156.8 (C)
2	132.8 (C)	137.2 (C)	138.3 (C)	137.6 (C)
3	125.2 (CH)	127.6 (CH)	127.9 (CH)	127.7 (CH)
4	121.9 (CH)	121.5 (CH)	121.3 (CH)	121.5 (CH)
5	131.9 (C)	133.5 (C)	131.0 (C)	132.5 (C)
6	117.0 (CH)	118.6 (CH)	118.3 (CH)	118.6 (CH)
7	169.7 (C)	171.3 (C)	168.5 (C)	169.9 (C)
8	74.9 (C)	77.8 (C)	77.8 (C)	77.7 (C)
9	38.1 (CH_2_)	43.3 (CH_2_)	43.9 (CH_2_)	43.4 (CH_2_)
10	18.3 (CH_2_)	23.0 (CH_2_)	19.9 (CH_2_)	22.4 (CH_2_)
11	36.5 (CH_2_)	35.4 (CH_2_)	45.0 (CH_2_)	34.7 (CH_2_)
12	100.9 (C)	40.9 (CH)	71.4 (C)	33.7 (CH)
13	28.6 (CH_3_)	181.6 (C)	29.2 (CH_3_)	17.1 (CH_3_)
14	26.9 (CH_3_)	17.7 (CH_3_)	29.1 (CH_3_)	70.5 (CH_2_)
15	-	28.9 (CH_3_)	28.8 (CH_3_)	28.8 (CH_3_)
16	-	-	52.5 (CH_3_)(COO*CH_3_)*	173.1 (C)(CO*CH_3_*)
CO*CH_3_*	-	-	-	20.8 (CH_3_)

**Table 2:** The ^1^H-NMR data of compounds **1**–**4** (400 MHz, CD_3_OD-*d_4_*).

No.	1	2	3	4
3	7.16 (d, 8.0, 1H)	7.24 (d, 8.2, 1H)	7.30 (d, 8.2, 1H)	7.27 (d, 8.0, 1H)
4	7.50 (dd, 1.7, 8.0, 1H)	7.43 (d, 8.4, 1H)	7.44 (dd, 1.8, 8.2,1H)	7.44 (d, 8.0, 1H)
5	-	-	-	-
6	7.34 (d, 1.7, 1H)	7.36 (br s, 1H)	7.36 (d, 1.8, 1H)	7.38 (br s, 1H)
7	-	-	-	-
8	-	-	-	-
9	1.81 (td, 4.2, 13.3, 1H)1.70 (m, 1H)	1.99 (m, 1H)1.81 (m, 1H)	1.98 (m, 1H)1.80 (m, 1H)	1.96(m, 1H)1.81(m, 1H)
10	1.62 (m, 1H)1.45 (m, 1H)	1.35 (m, 1H)1.21 (m, 1H)	1.39 (m, 1H)1.25(m, 1H)	1.29 (m,2H)
11	1.97 (br d, 13.3, 1H)1.73 (m, 1H)	1.58 (m, 1H)1.34 (m, 1H)	1.39 (m, 2H)	1.35 (m, 1H)1.11 (m, 1H)
12	-	2.34 (m, 1H)	-	1.72 (m, 1H)
13	1.50 (s, 3H)	-	1.01 (s, 3H)	0.86 (d, 6.8, 3H)
14	1.60 (s, 3H)	1.07 (d, 7.0, 3H)	1.01 (s, 3H)	3.88 (m, 1H)3.80 (m, 1H)
15	-	1.59 (s, 3H)	1.60 (s, 3H)	1.60 (s, 3H)
COO*CH_3_*	-	-	3.88 (s, 3H)	-
CO*CH_3_*	-	-	-	2.00 (s, 3H)

**Table 3:** The biological activity assays data of **1**–**12**.

No.	Anticanceractivity (IC_50_ in μg/mL)				Antimicobial Activity(MIC in μg/mL)
	A549	Caski	HepG2	MCF-7	*Erwinia carotovora*
1	54.2	58.3	80.3	90.6	15.2
2	65.4	75.3	90.8	57.4	35.6
3	67.3	73.5	69.5	64.4	40.5
4	85.6	85.5	74.4	88.7	50.8
5	72.2	92.2	75.7	65.3	64.3
6	90.6	78.2	75.8	80.4	68.9
7	82.5	90.4	60.5	77.5	75.2
8	64.4	65.6	59.2	64.1	85.2
9	89.7	83.5	76.2	79.1	68.8
10	94.2	90.4	88.2	96.8	75.3
11	100.1	95.3	100.5	78.9	65.4
12	55.6	85.2	78.8	65.4	50.9

## Supplementary Materials

Supplementary materials are available on line.
