# Supplementary material for: Aspergoterpenins A–D: Four New Antimicrobial Bisabolane Sesquiterpenoid Derivatives from an Endophytic Fungus Aspergillus versicolor
Source: Molecules. 2018 May 28;23(6):1291. doi: 10.3390/molecules23061291 (PMC6100428; doi:10.3390/molecules23061291)
Supplement: Supplementary file 1 [file molecules-23-01291-s001.pdf]

Aspergoterpenins A-D, Four New Antimicrobial bisabolane sesquiterpenoid Derivatives from an Endophytic Fungus *Aspergillus versicolor*

Zhi-Yong Guo[1], Ming-Hui Tan[1], Cheng-Xiong Liu[1], Meng-Meng Lv[1], Zhang-Shuang Deng[1,*], Fei Cao[2,*], Kun Zou[1], Peter Proksch[3]

Zhi-Yong Guo[1,3], Ming-Hui Tan[1], Cheng-Xiong Liu[1], Meng-Meng Lv[1], Fei Cao[2,*], Zhang-Shuang Deng[1,3,*], Kun Zou[1], Peter Proksch[4]

[1]Hubei Key Laboratory of Natural Product Research and Development (China Three Gorges University), College of Biological and Pharmaceutical Sciences, China Three Gorges University, Yichang, 443002, People's Republic of China

[2] College of Pharmaceutical Sciences, Key Laboratory of Pharmaceutical Quality Control of Hebei Province, Key Laboratory of Medicinal Chemistry and Molecular Diagnostics of Education Ministry of China, Hebei University, Baoding 071002, People's Republic of China.

[3] Hubei Engineering Research Center for Three Gorges Regional Plant Breeding，China Three Gorges University, Yichang 443002, People's Republic of China.

[4] Institute of Pharmaceutical Biology and Biotechnology, Heinrich-Heine-Universität Düsseldorf, Universitätsstrasse 1, 40225 Düsseldorf, Germany

* Correspondence: dzs163@163.com (Zhang-Shuang Deng) and caofei542927001@163.com (Fei Cao), Tel/Fax: + 86 - 717 – 6397478

S1: the HRESIMS of compound **1**(Aspergoterpenin A)

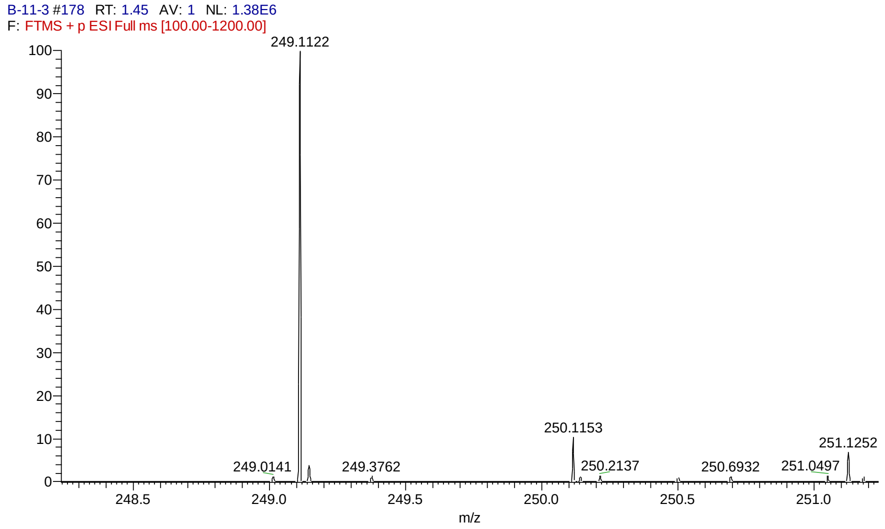

S2: the NMR spectra of compound **1** (Aspergoterpenin A)

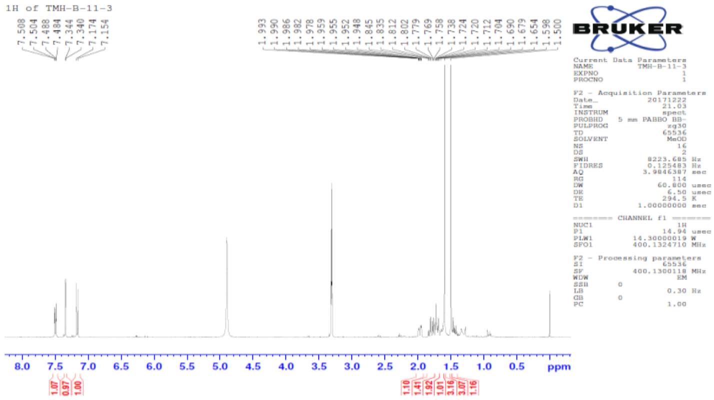
1H of TMH-B-11-3
7.508
7.504
7.488
7.484
7.344
7.340
7.174
7.154
1.993
1.990
1.986
1.982
1.978
1.959
1.955
1.952
1.948
1.845
1.835
1.812
1.802
1.779
1.769
1.758
1.738
1.724
1.720
1.712
1.704
1.690
1.679
1.654
1.598
1.500
BRUKER
Current Data Parameters
NAME TMH-B-11-3
EXPNO 1
PROCNO 1
F2 - Acquisition Parameters
Date_ 20171222
Time 21.03
INSTRUM spect
PROBHD 5 mm PABBO BB-
PULPROG zg30
TD 65536
SOLVENT MeOD
NS 16
DS 2
SWH 8223.685 Hz
FIDRES 0.125483 Hz
AQ 3.9846387 sec
RG 114
DW 60.800 usec
DE 6.50 usec
TE 294.5 K
D1 1.00000000 sec
======== CHANNEL f1 ========
NUC1 1H
P1 14.94 usec
PLW1 14.30000019 W
SFO1 400.1324710 MHz
F2 - Processing parameters
SI 65536
SF 400.1300118 MHz
WDW EM
SSB 0
LB 0.30 Hz
GB 0
PC 1.00
8.0 7.5 7.0 6.5 6.0 5.5 5.0 4.5 4.0 3.5 3.0 2.5 2.0 1.5 1.0 0.5 ppm
1.07
0.97
1.00
1.10
1.41
1.92
1.01
3.16
3.07
1.16

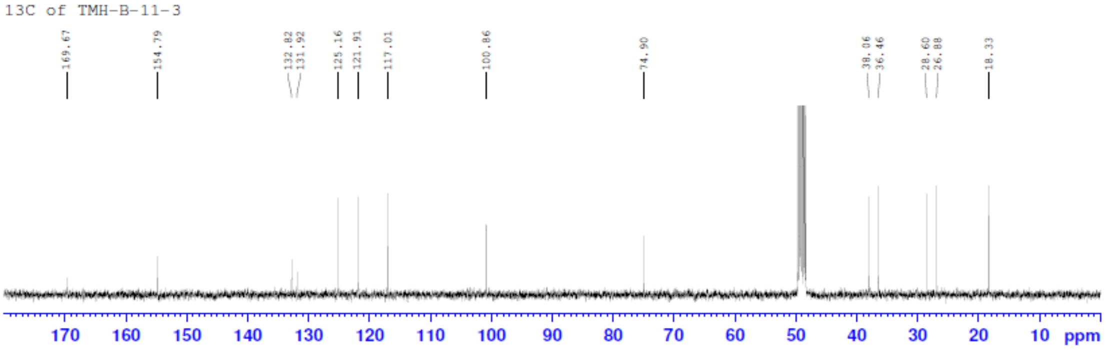
13C of TMH-B-11-3
169.67
154.79
132.82
131.92
125.16
121.91
117.01
100.86
74.90
38.06
36.46
28.60
26.88
18.33
170 160 150 140 130 120 110 100 90 80 70 60 50 40 30 20 10 ppm

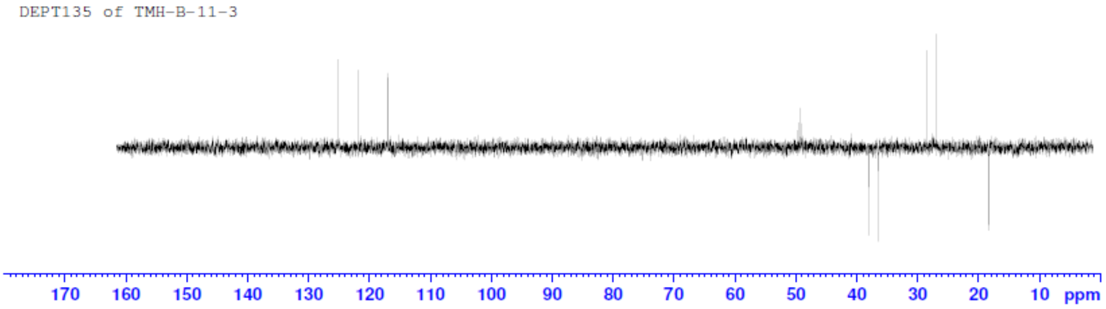
DEPT135 of TMH-B-11-3
170 160 150 140 130 120 110 100 90 80 70 60 50 40 30 20 10 ppm

COSY of TMH-B-11-3

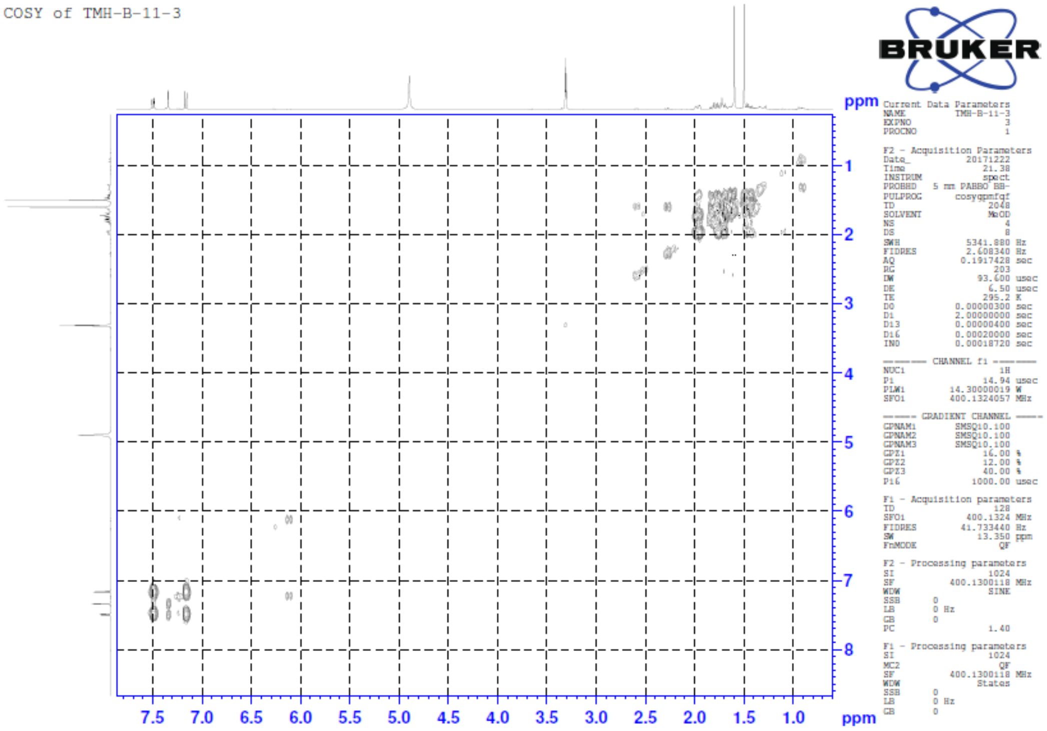

HSQC of TMH-B-11-3

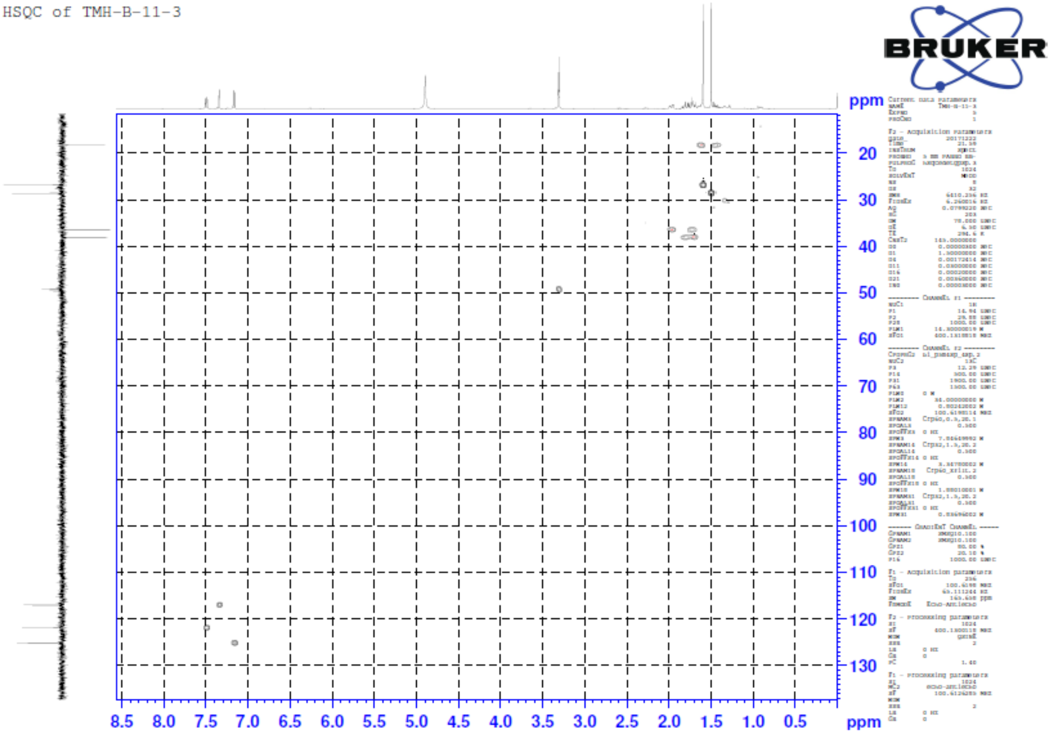

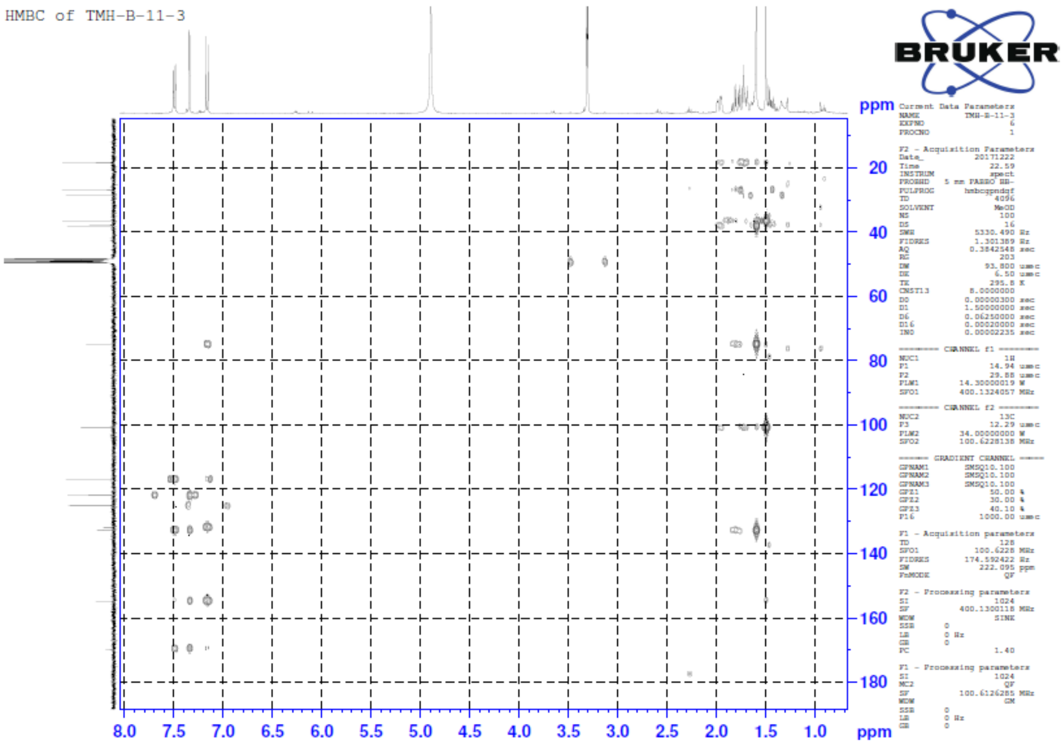

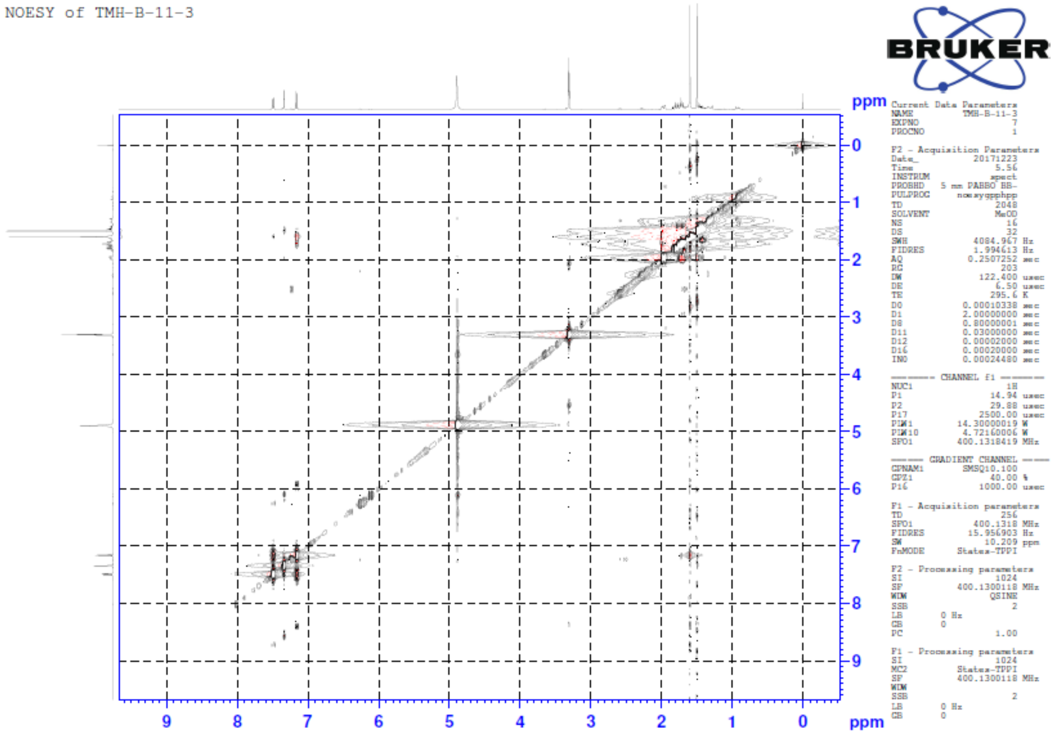

S2: the HRESIMS of compound **2** (Aspergoterpenin B)

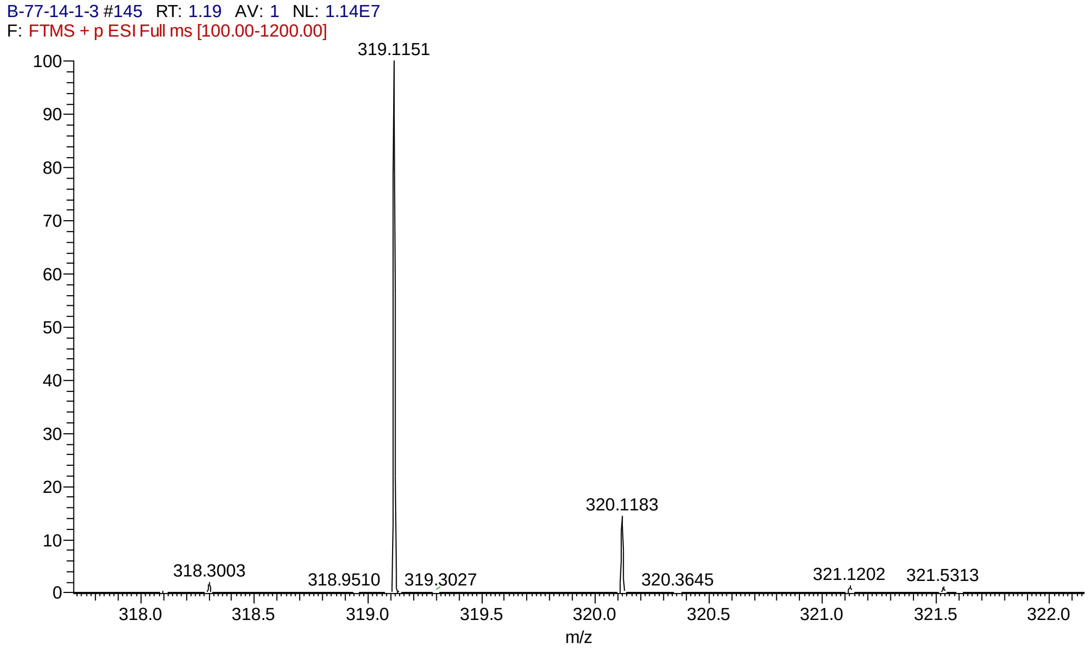

S3: the NMR spectra of compound **2** (Aspergoterpenin B)

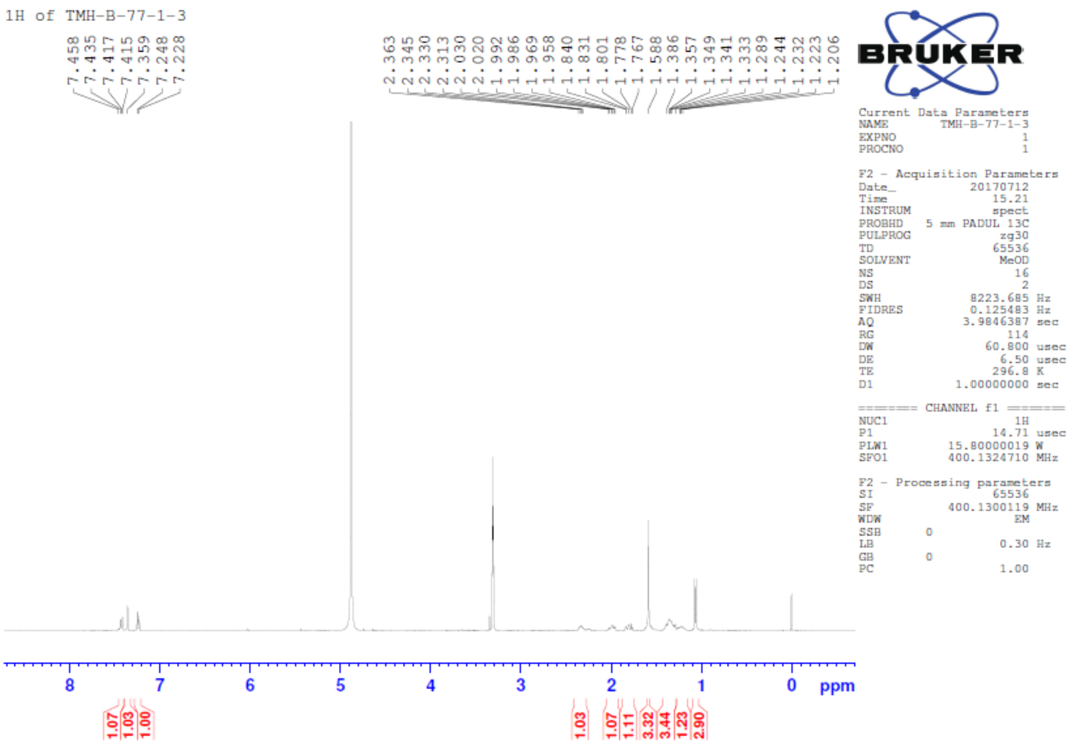

13C of TMH-B-77-1-3

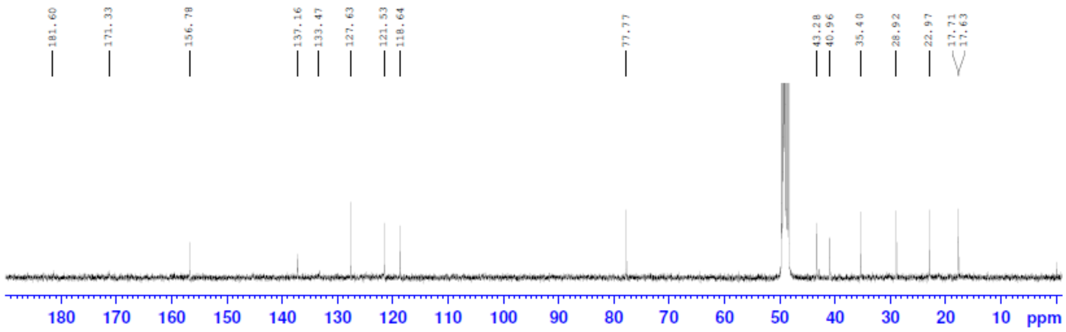

DEPT135 of TMH-B-77-1-3

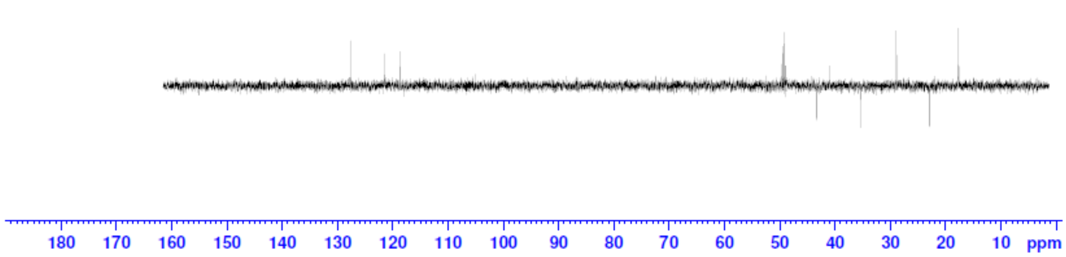

COSY of TMH-B-77-1-3

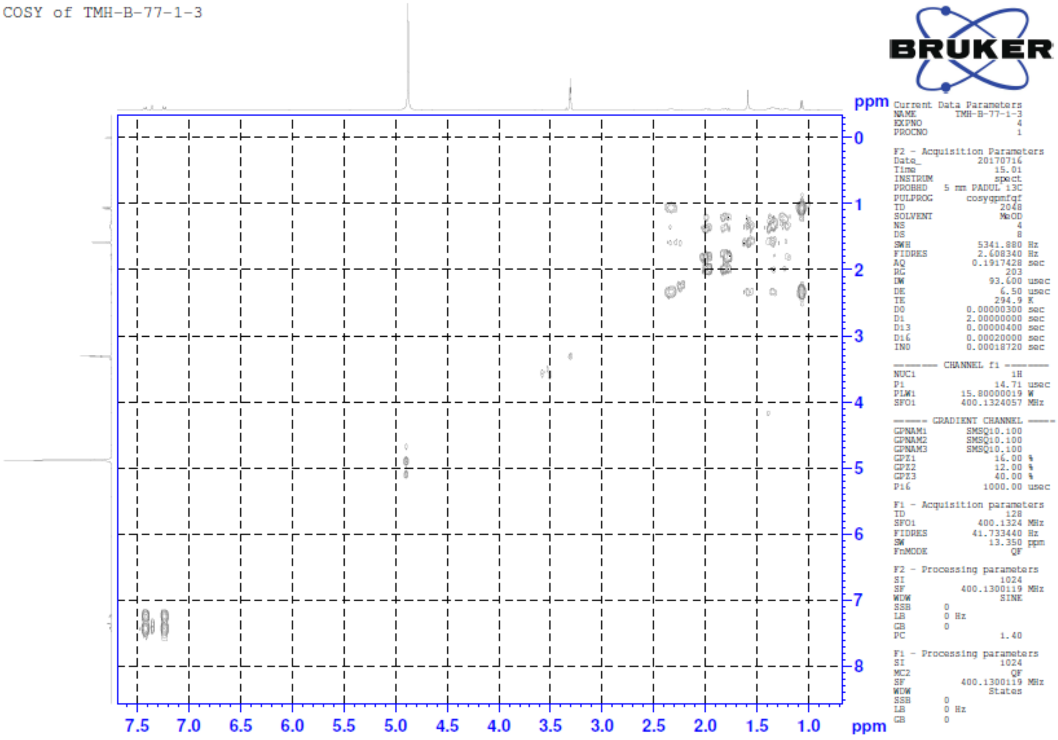


```
Current Data Parameters
NAME         TMH-B-77-1-3
EXPNO                   4
PROCNO                  1

F2 - Acquisition Parameters
Date_            20170716
Time                15.01
INSTRUM             spect
PROBHD   5 mm PADUL 13C
PULPROG         cosygpmfqf
TD                   2048
SOLVENT              MeOD
NS                      4
DS                      8
SWH              5341.880 Hz
FIDRES           2.608340 Hz
AQ              0.1917428 sec
RG                    203
DW                 93.600 usec
DE                   6.50 usec
TE                  294.9 K
D0             0.00000300 sec
D1             2.00000000 sec
D13            0.00000400 sec
D16            0.00020000 sec
IN0            0.00018720 sec

====== CHANNEL f1 ======
NUC1                   1H
P1                  14.71 usec
PLW1          15.80000019 W
SFO1          400.1324057 MHz

==== GRADIENT CHANNEL ====
GPNAM1         SMSQ10.100
GPNAM2         SMSQ10.100
GPNAM3         SMSQ10.100
GPZ1                16.00 %
GPZ2                12.00 %
GPZ3                40.00 %
P16               1000.00 usec

F1 - Acquisition parameters
TD                    128
SFO1             400.1324 MHz
FIDRES          41.733440 Hz
SW                 13.350 ppm
FnMODE                 QF

F2 - Processing parameters
SI                   1024
SF            400.1300119 MHz
WDW                  SINE
SSB      0
LB       0 Hz
GB       0
PC                   1.40

F1 - Processing parameters
SI                   1024
MC2                    QF
SF            400.1300119 MHz
WDW                States
SSB      0
LB       0 Hz
GB       0
```

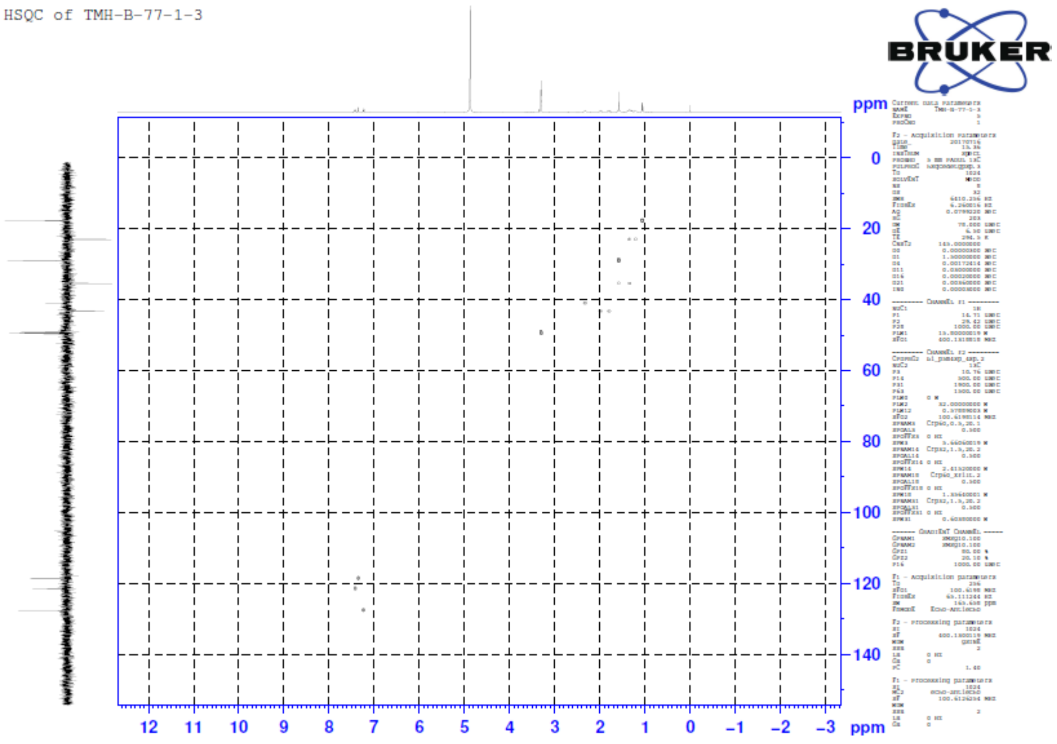

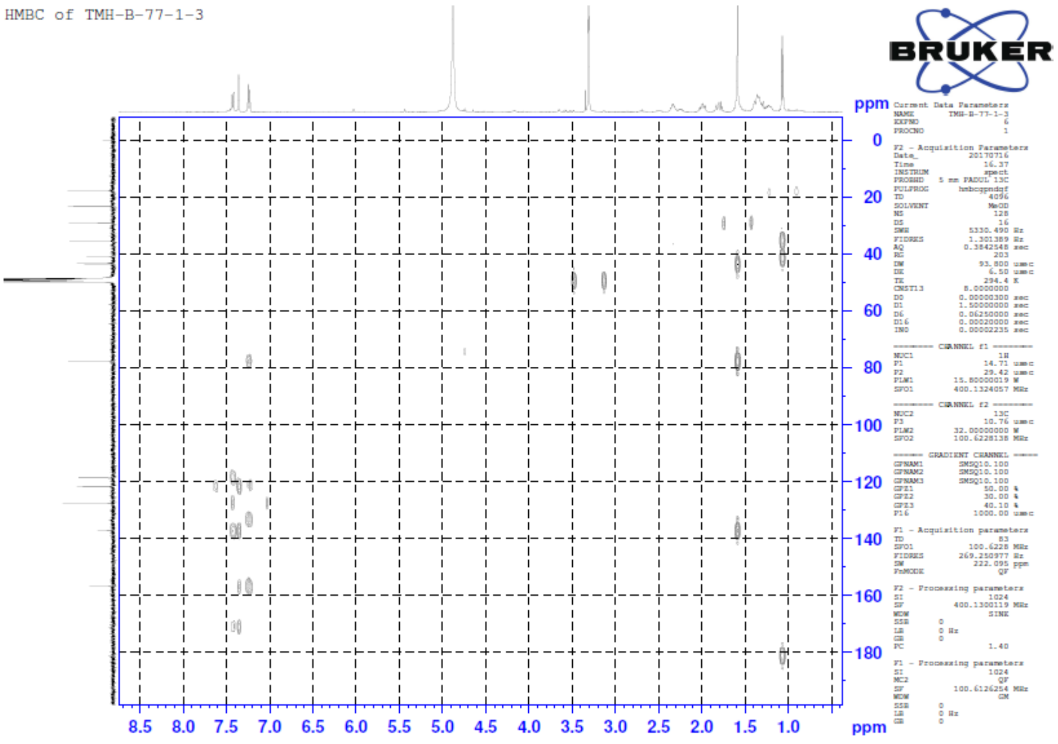

S4: the HRESIMS of compound **3** (Aspergoterpenin C)

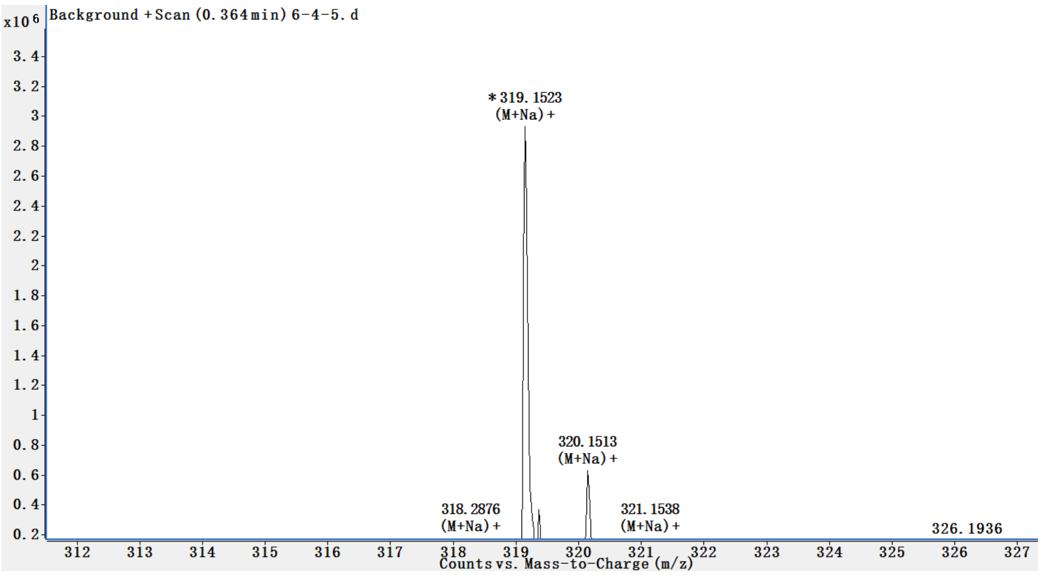

S5: the NMR spectra of compound **3** (Aspergoterpenin C)

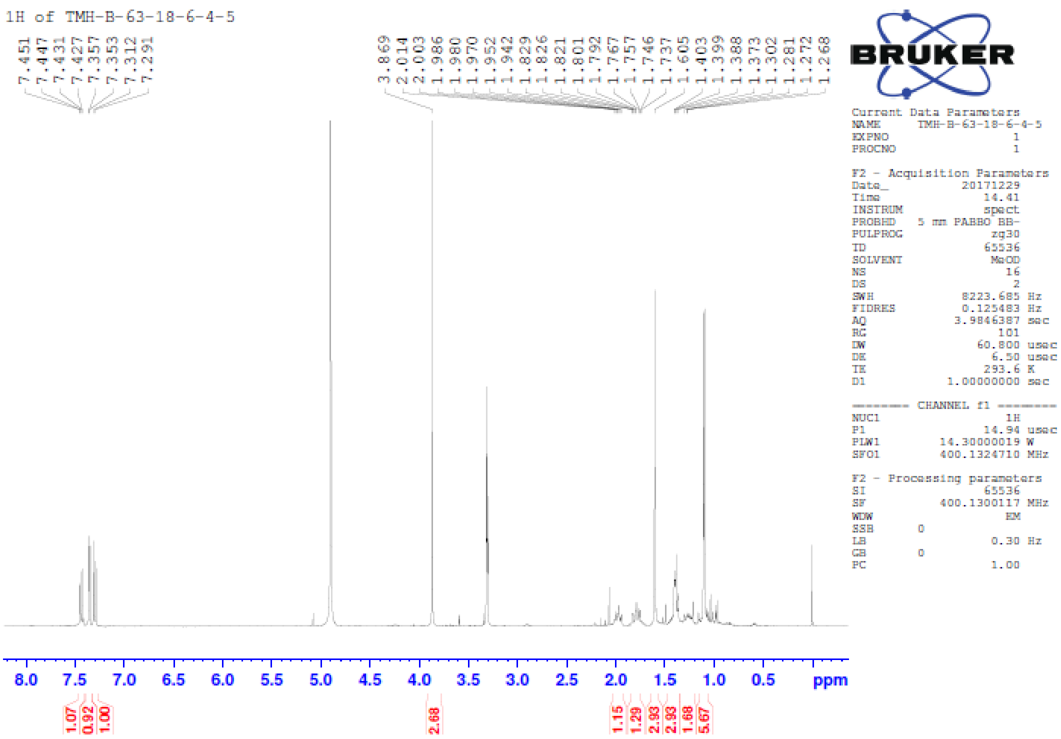

13C of TMH-B-63-18-6-4-5

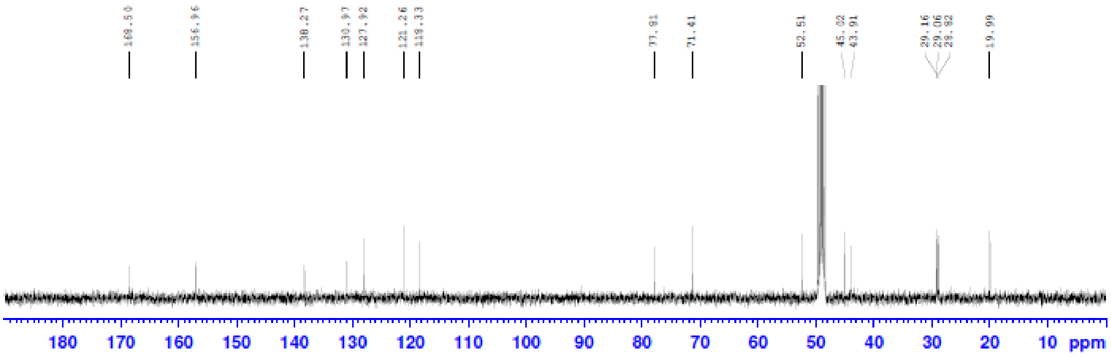

DEPT135 of TMH-B-63-18-6-4-5

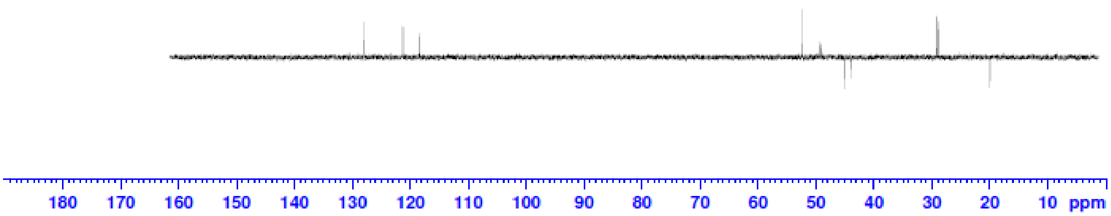

COSY of TMH-B-63-18-6-4-5

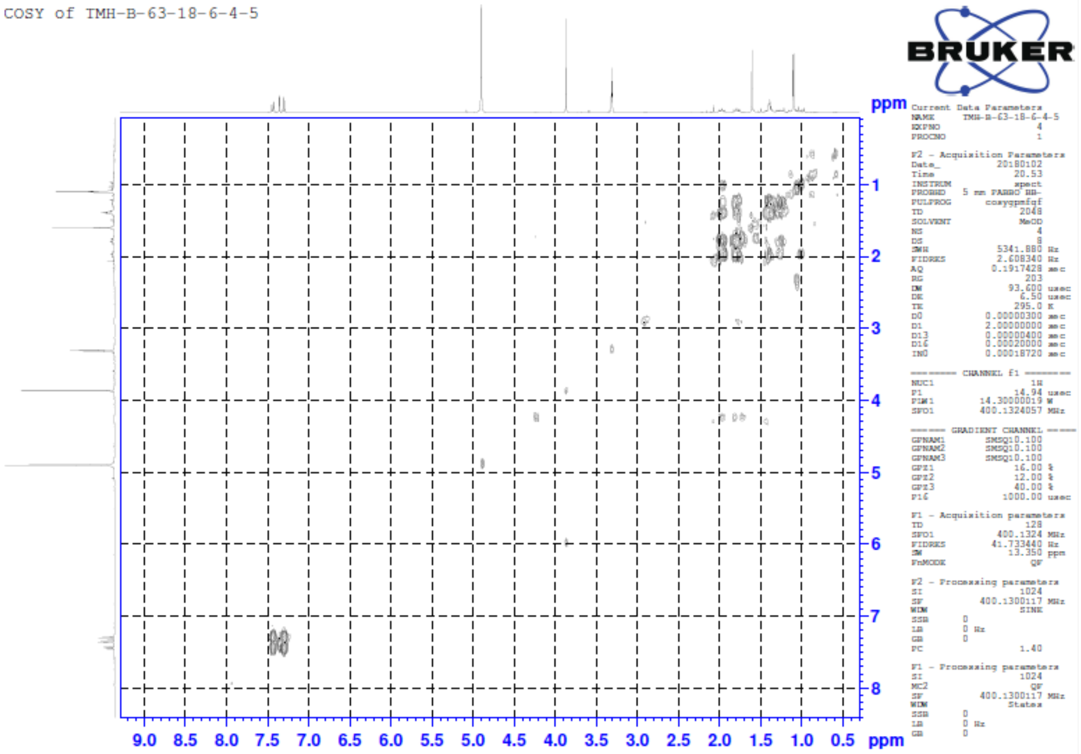

HSQC of TMH-B-63-18-6-4-5

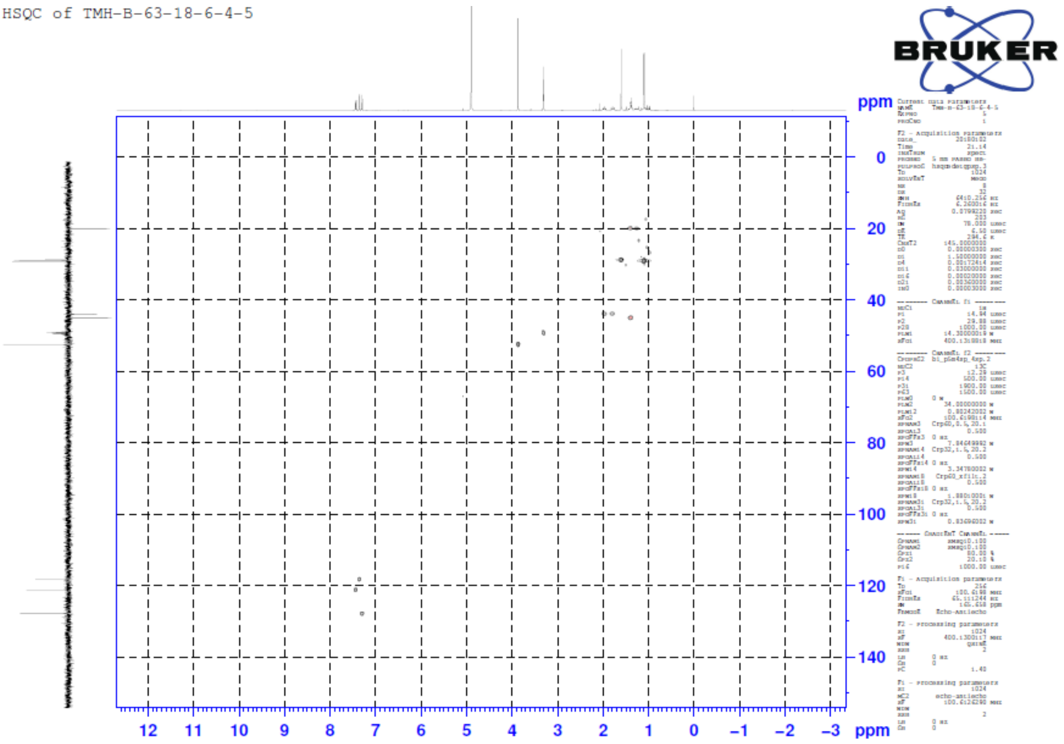

HMBC of TMH-B-63-18-6-4-5

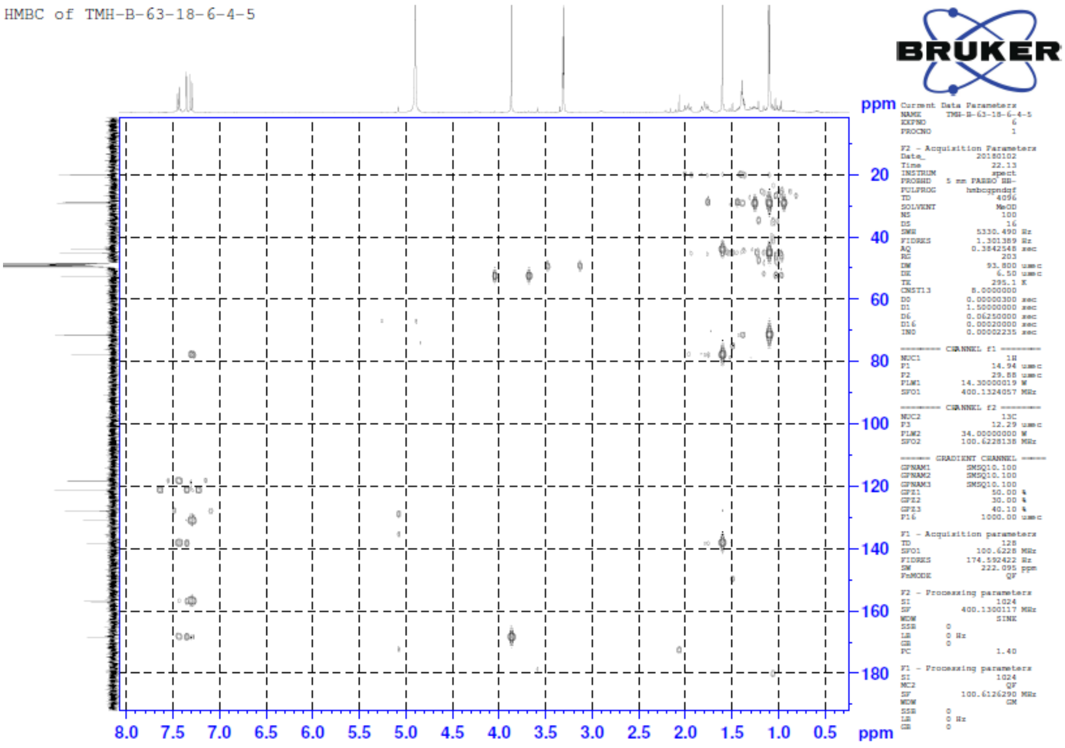

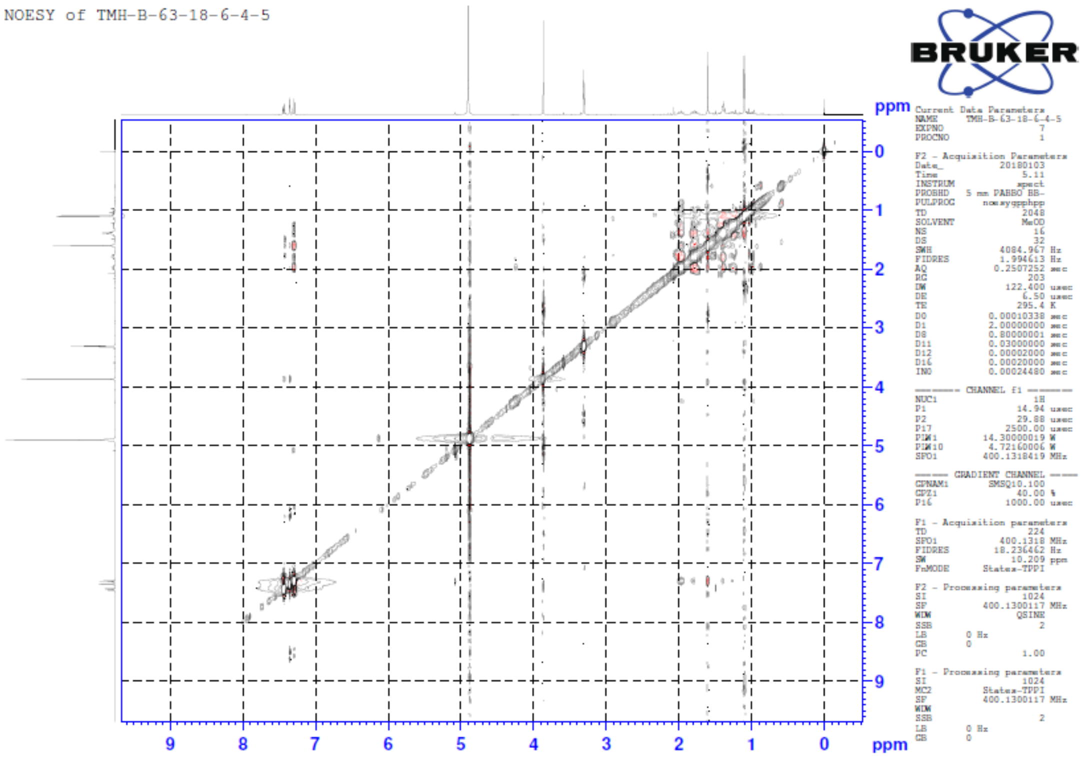

S6: the HRESIMS of compound **4** (Aspergoterpenin D)

S7: the NMR spectra of compound **4** (Aspergoterpenin D)

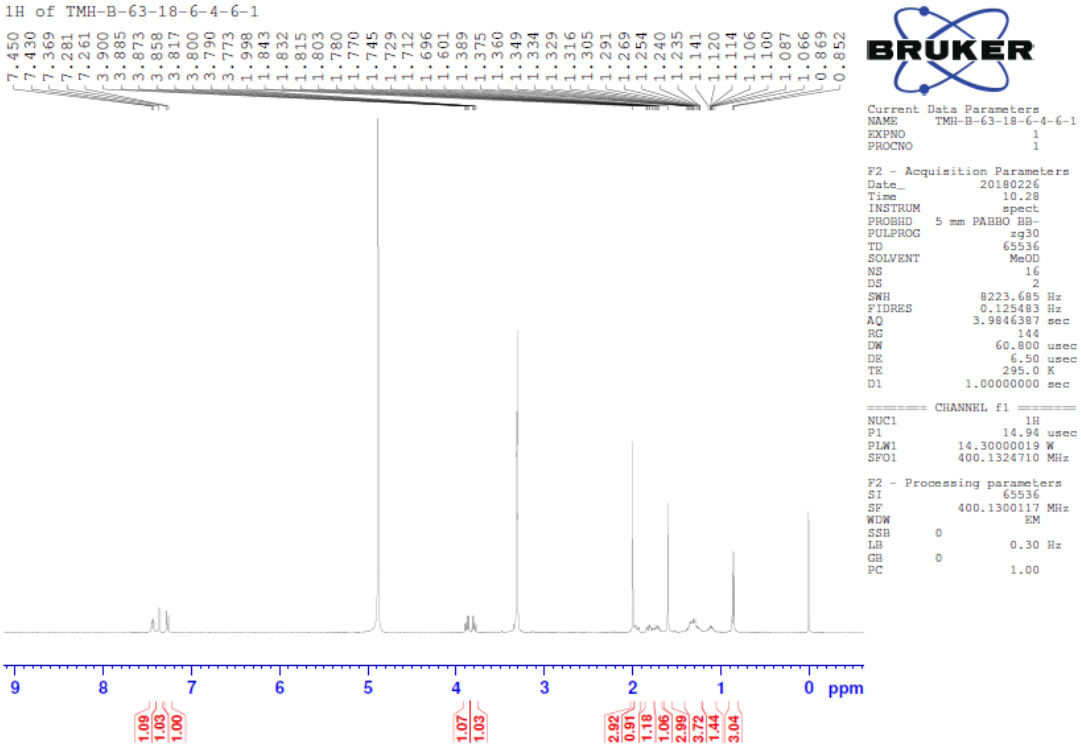

13C of TMH-B-63-18-6-4-6-1

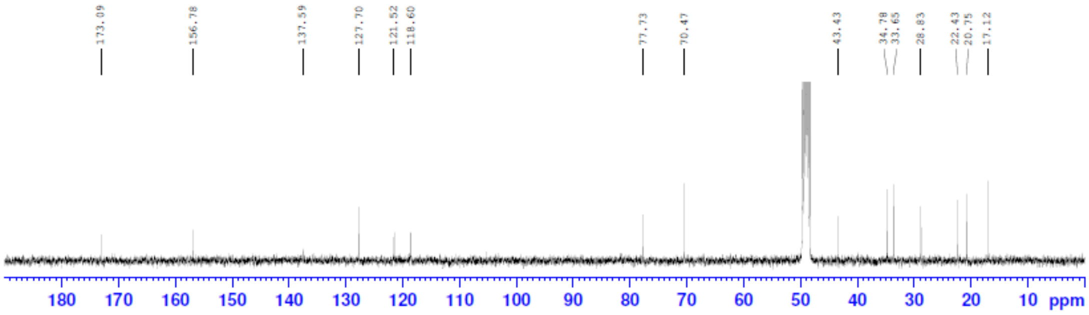

DEPT135 of TMH-B-63-18-6-4-6-1

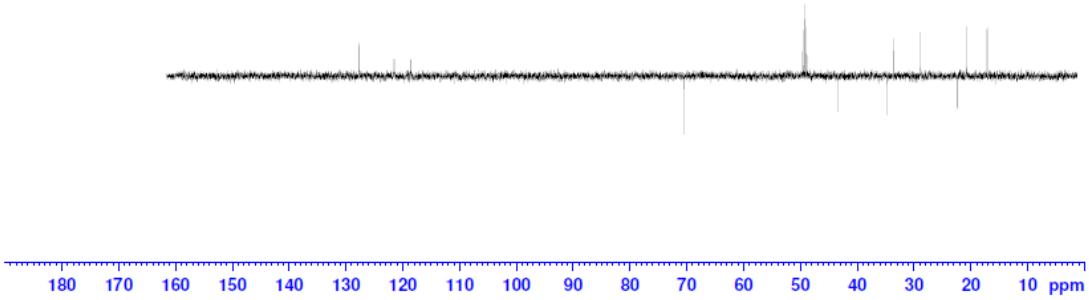

COSY of TMH-B-63-18-6-4-6-1

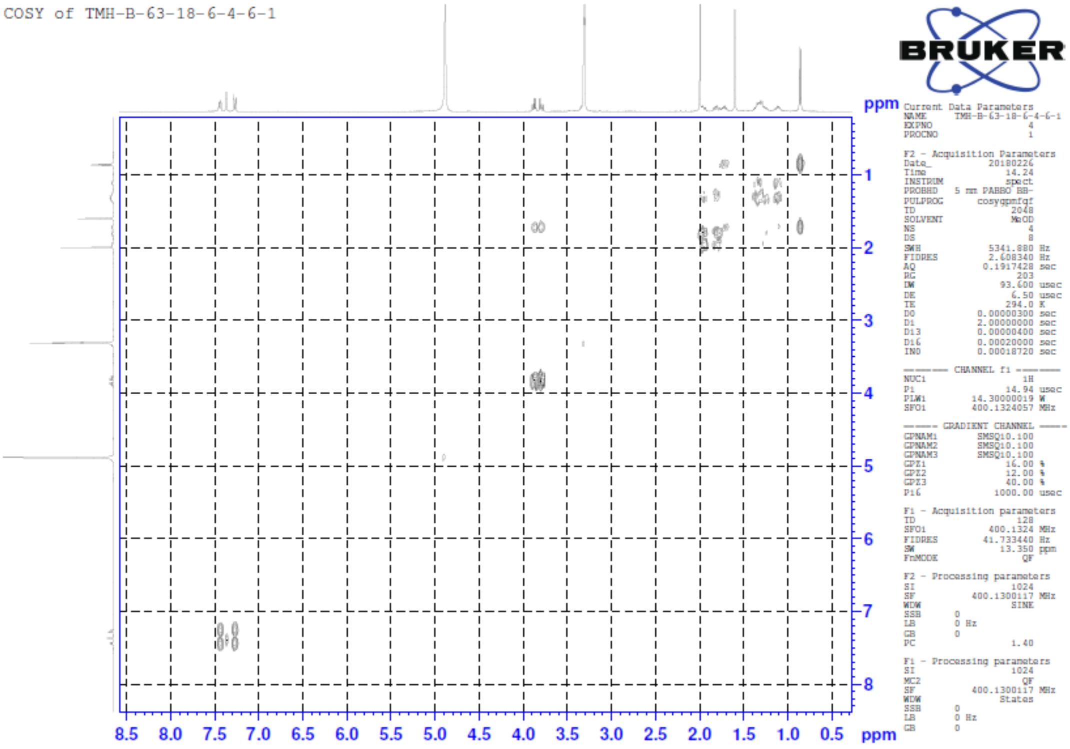

Current Data Parameters
NAME TMH-B-63-18-6-4-6-1
EXPNO 4
PROCNO 1

F2 - Acquisition Parameters
Date_ 20180226
Time 14.24
INSTRUM spect
PROBHD 5 mm PABBO BB-
PULPROG cosygpmfqf
TD 2048
SOLVENT MeOD
NS 4
DS 8
SWH 5341.880 Hz
FIDRES 2.608340 Hz
AQ 0.1917428 sec
RG 203
DW 93.600 usec
DE 6.50 usec
TE 294.0 K
D0 0.00000300 sec
D1 2.00000000 sec
D13 0.00000400 sec
D16 0.00020000 sec
IN0 0.00018720 sec

====== CHANNEL f1 ======
NUC1 1H
P1 14.94 usec
PLW1 14.30000019 W
SFO1 400.1324057 MHz

===== GRADIENT CHANNEL =====
GPNAM1 SMSQ10.100
GPNAM2 SMSQ10.100
GPNAM3 SMSQ10.100
GPZ1 16.00 %
GPZ2 12.00 %
GPZ3 40.00 %
P16 1000.00 usec

F1 - Acquisition parameters
TD 128
SFO1 400.1324 MHz
FIDRES 41.733440 Hz
SW 13.350 ppm
FnMODE QF

F2 - Processing parameters
SI 1024
SF 400.1300117 MHz
WDW SINE
SSB 0
LB 0 Hz
GB 0
PC 1.40

F1 - Processing parameters
SI 1024
MC2 QF
SF 400.1300117 MHz
WDW States
SSB 0
LB 0 Hz
GB 0

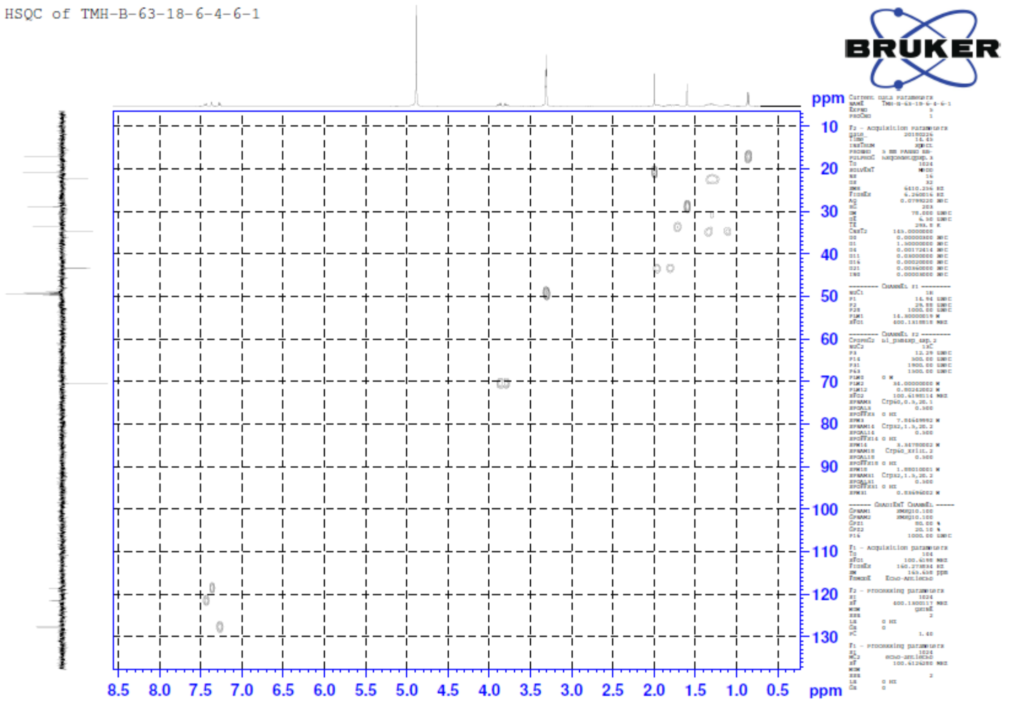
HSQC of TMH-B-63-18-6-4-6-1
BRUKER
ppm
10
20
30
40
50
60
70
80
90
100
110
120
130
8.5 8.0 7.5 7.0 6.5 6.0 5.5 5.0 4.5 4.0 3.5 3.0 2.5 2.0 1.5 1.0 0.5 ppm

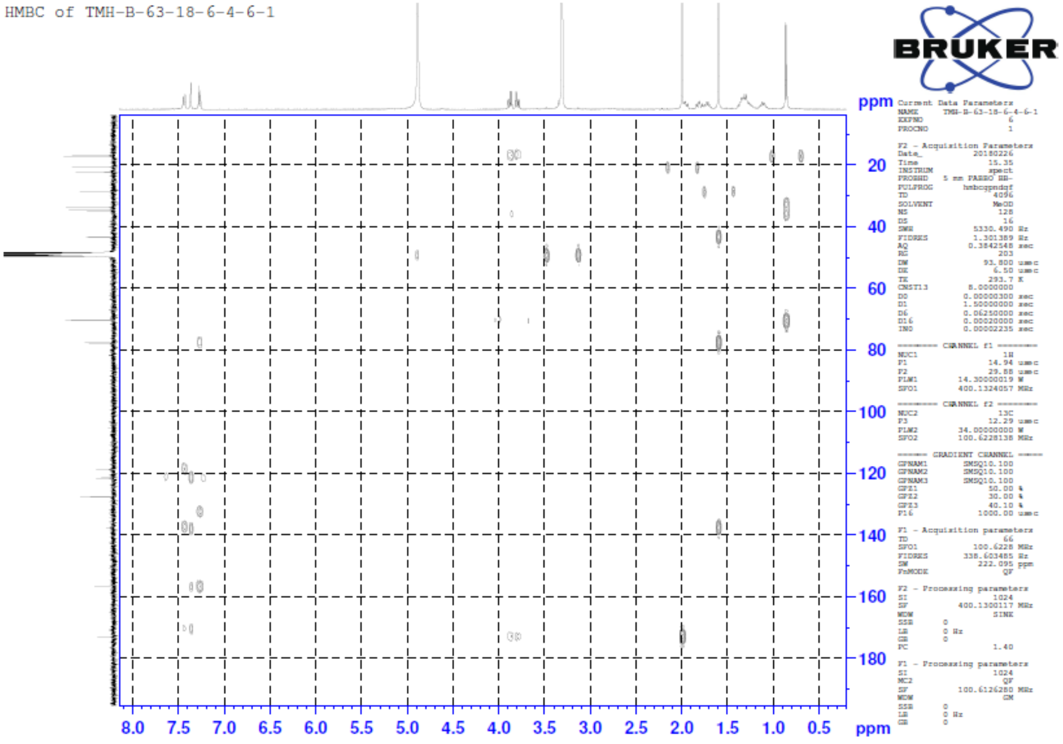
HMBC of TMH-B-63-18-6-4-6-1
BRUKER
ppm
20
40
60
80
100
120
140
160
180
8.0 7.5 7.0 6.5 6.0 5.5 5.0 4.5 4.0 3.5 3.0 2.5 2.0 1.5 1.0 0.5 ppm
